# Uncommon Presentation of Mastocytosis: Focus on the Xanthelasmoid Variant

**DOI:** 10.7759/cureus.90426

**Published:** 2025-08-18

**Authors:** Salma Kozmane, Hanane Baybay, Aya Tadlaoui, Zakia Douhi, FatimaZahra Mernissi

**Affiliations:** 1 Dermatology Department, Hassan II University Hospital, Fez, MAR

**Keywords:** child, dermoscopy, mastocytosis, multiple juvenile xanthogranulomas, xanthelasmoid mastocytosis

## Abstract

Xanthelasmoid mastocytosis (XM) is a rare form of diffuse cutaneous mastocytosis whose clinical presentation may resemble multiple juvenile xanthogranulomas. We report the case of a two-year-old child who presented with multiple yellowish papules and nodules, with a firm to elastic consistency, symmetrically distributed over the face, the base of the neck, trunk, and limbs. Dermoscopy and histology confirmed the diagnosis of XM. The patient was successfully treated with H1-antihistamines and high-potency topical corticosteroids. This case highlights the importance of considering XM as a rare variant of diffuse cutaneous mastocytosis that may be confused with multiple juvenile xanthogranulomas.

## Introduction

Mastocytosis is a rare disease that presents in two-thirds of cases in children under the age of two years. It is associated with the proliferation of mast cells in one or more organs due to mutations in the proto-oncogene c-kit. Mastocytosis generally develops during infancy or early adulthood. In children, it most often remains restricted to the skin, although systemic symptoms may arise from mast cell mediator release, even in the absence of systemic infiltration [1]. In children, the clinical variants that have been reported are, in descending order, as follows: urticaria pigmentosa, papulonodular form, mastocytoma, and diffuse cutaneous mastocytosis [2]. We report a new case of xanthelasmoid mastocytosis (XM), a rare form of diffuse cutaneous mastocytosis, whose clinical presentation may be relevant in the differential diagnosis of multiple juvenile xanthogranuloma.

## Case presentation

**Figure 1:**
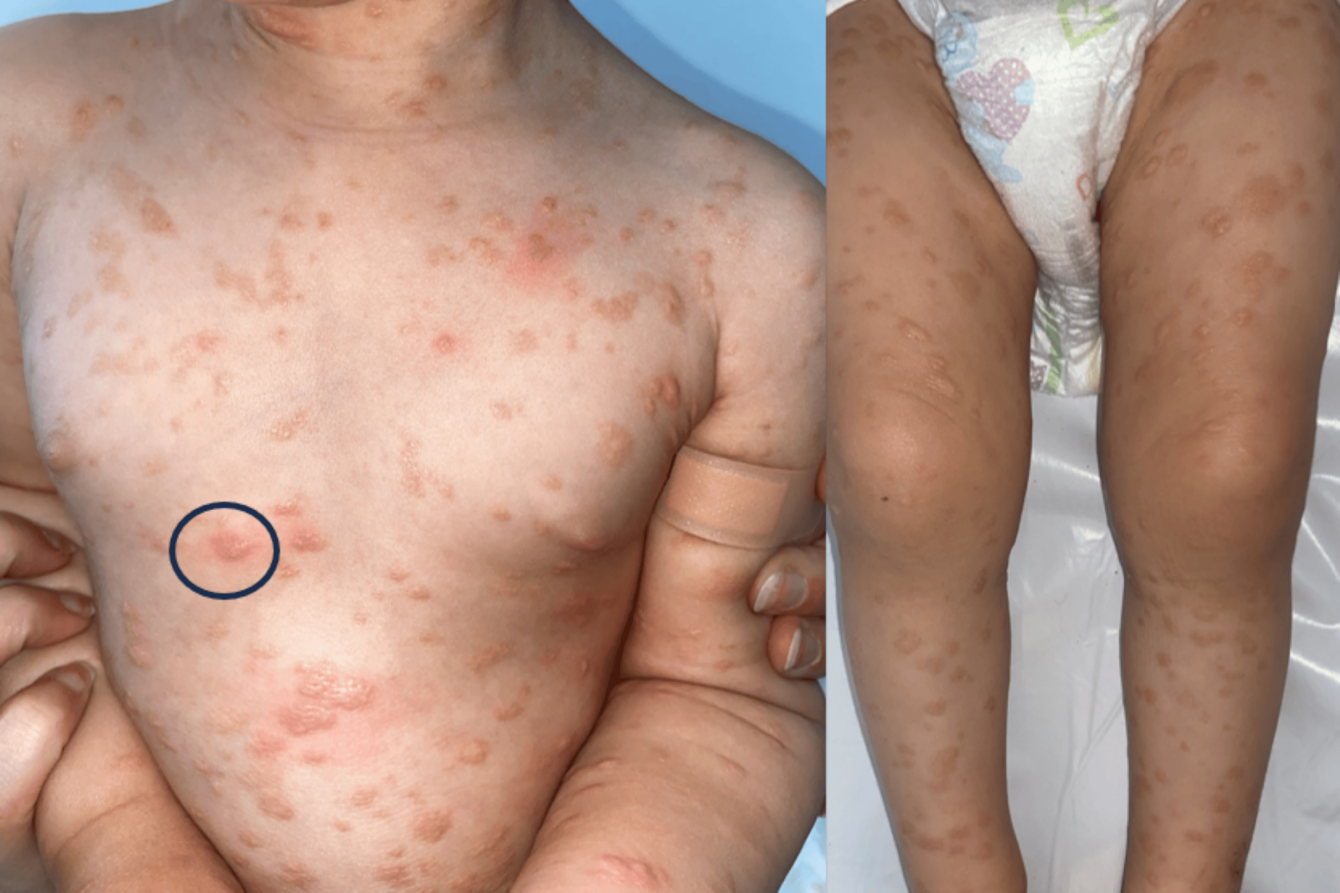
Multiple yellowish papules and nodules, symmetrically distributed on the trunk and upper and lower limbs with positive Darier’s sign (blue circle).

We report the case of a two-year-old child, born to non-consanguineous parents, who presented with diffuse cutaneous lesions over the entire body for the past five months, without associated gastrointestinal symptoms, but with intermittent pruritus triggered by heat. There were no episodes of flushing or diarrhea. Clinical examination revealed multiple yellowish papules and nodules, with a firm to elastic consistency, symmetrically distributed over the face, the base of the neck, trunk, and limbs (Figure 1).

**Figure 2:**
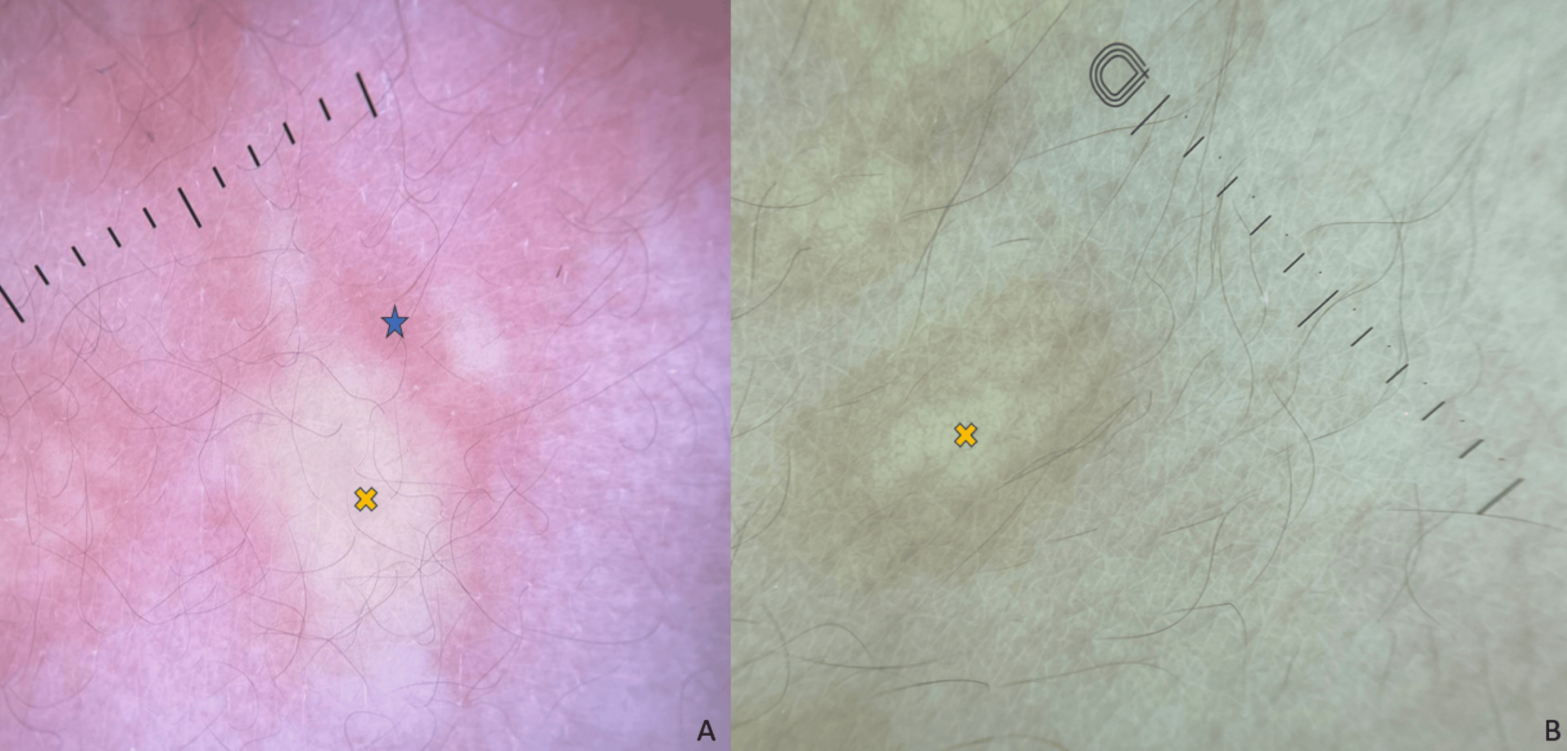
Dermoscopic features: erythematous background (blue star) with a central homogeneous yellow pattern (yellow cross) after rubbing (A) and before rubbing (B).

Darier’s sign was positive. Dermoscopy showed a central homogeneous yellow pattern in some areas and a peripheral pigmented network (Figure 2A, 2B).

**Figure 3:**
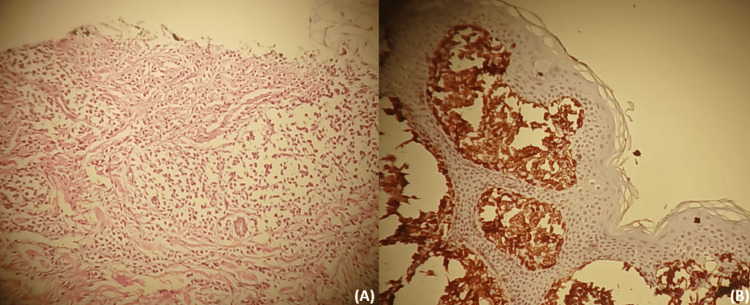
A: Hematoxylin and eosin stain showing a collection of mast cells in cords and nests in dermis. B: Immunohistochemistry stain showing mast cells positive for CD 117.

The child had normal growth parameters, and the remainder of the physical examination was unremarkable. The differential diagnoses considered were XM and multiple juvenile xanthogranuloma. Histological examination revealed a dense, sheet-like infiltrate involving both superficial and deep dermis; the cells exhibited a single oval-shaped nucleus and granular cytoplasm. Immunohistochemical staining with CD117 was positive, confirming the diagnosis of cutaneous mastocytosis (Figure 3A, 3B).

Plasma tryptase levels were within normal range. The final diagnosis was XM. Management included educating the parents about the natural course and prognosis of the disease, as well as the importance of avoiding known triggering factors, including certain medications that can induce mast cell degranulation and exacerbate skin lesions. Moreover, administration of H1-antihistamines and the application of a compounded preparation containing a high-potency topical corticosteroid. The evolution was marked by partial regression of the lesions after six months of follow-up.

## Discussion

Mastocytosis is a heterogeneous group of disorders characterized by the abnormal proliferation of mast cells, which may be immature or mature, within one or more organs. These are rare and orphan diseases, with an estimated incidence of approximately two cases per 300,000 individuals per year. While most cases occur sporadically, familial forms have also been described. The clinical manifestations result from either the excessive release of mast cell mediators or the accumulation of mast cells in affected tissues. There are various clinical manifestations, including urticaria pigmentosa, papulonodular urticaria, mastocytoma, and diffuse cutaneous mastocytosis [2]. In mastocytosis, both local and systemic manifestations result from the excessive release of mast cell mediators, including histamine, leukotrienes, proteases, and heparin. Clinical symptoms differ among patients and may include pruritus, dyspnea, flushing, blister formation, bone pain, and gastrointestinal complaints such as vomiting, diarrhea, and epigastric discomfort [3].

XM is a very rare form classified as papulonodular mastocytosis. The first case was described by Tilbery in 1875 [4]. Its prevalence is difficult to estimate: to date, some 15 observations have been reported in the literature. Their clinical presentation is characterized by the presence of soft, yellow-brown papules or nodules of varying sizes. The triggers are typical of mastocytosis. Darier's signs often disappear, but in our case, it was present. Furthermore, this clinical form is characterized by the persistence of lesions after puberty without an increased risk of systemic compromise [5].

According to the World Health Organization criteria, the diagnosis of cutaneous mastocytosis is based on the presence of typical or atypical skin lesions, a positive Darier's sign, and the exclusion of other skin diseases. The diagnosis may be supported by one of two minor criteria: The presence of mast cell clusters (more than 15 mast cells per aggregate at high magnification or a single infiltrate of more than 20 mast cells per field) and/or detection of a point mutation in codon 816 of the KIT gene in affected skin tissue [2]. Diagnosis is made clinically and/or by skin biopsy with histological examination, and management is largely guided by clinical data. The benign nature of the condition in the vast majority of cases means that further investigations are not systematically necessary. It is estimated that, overall, in the course of mastocytosis, a predictive value for systemic damage would be 50% of cases with tryptase levels between 25 and 75 µg/ml and practically guaranteed if the level is above 75 µg/ml [2]. The normal tryptase levels in our patients’ plasma helped to assess this risk and had a good positive predictive value.

There are no curative treatments for mastocytosis in children. Management strategies will depend on the clinical form. Avoiding triggers is always advisable, like heat; sudden temperature changes; skin friction; scalp trauma; emotional stress; infections with fever; certain drugs like nonsteroidal anti-inflammatory drugs, morphine derivatives, and dextromethorphan; and various procedures including dental work, vaccinations, and surgery [6]. The use of high-potency topical corticosteroids can help alleviate pruritus, urticaria, and flushing. A combination of H1 and/or H2 antihistamines may also be considered to improve symptoms such as itching, gastrointestinal discomfort, and diarrhea [7]. In severe cases presenting with extensive bullous detachment, short-term systemic corticosteroid therapy, typically ranging from 0.5 to 1 mg/kg/day, depending on the clinical scenario, has shown favorable outcomes. In addition, tyrosine kinase inhibitors have demonstrated efficacy in a limited number of reported cases [5].

## Conclusions

Xanthelasma-like cutaneous mastocytosis is an exceptionally rare variant of diffuse cutaneous mastocytosis, characterized by yellowish infiltrated lesions that can mimic xanthogranulomatous disorders such as juvenile xanthogranuloma. Accurate diagnosis relies on histopathological examination, which demonstrates dense mast cell infiltration in the deep dermis, confirmed by toluidine blue color and CD117 marker. Recognizing this atypical presentation is essential to avoid misdiagnosis and ensure appropriate management. 
